# Attention Autoencoder for Generative Latent Representational Learning in Anomaly Detection

**DOI:** 10.3390/s22010123

**Published:** 2021-12-24

**Authors:** Ariyo Oluwasanmi, Muhammad Umar Aftab, Edward Baagyere, Zhiguang Qin, Muhammad Ahmad, Manuel Mazzara

**Affiliations:** 1 School of Information and Software Engineering, University of Electronic Science and Technology of China, Chengdu 610054, China; ariyo@uestc.edu.cn (A.O.); ybaagyere@uds.edu.gh (E.B.); 2 Department of Computer Science, National University of Computer and Emerging Sciences, Islamabad, Chiniot-Faisalabad Campus, Chiniot 35400, Pakistan; umar.aftab@nu.edu.pk (M.U.A.); mahmad00@gmail.com (M.A.); 3 Institute of Software Development and Engineering, Innopolis University, Innopolis 420500, Russia; m.mazzara@innopolis.ru

**Keywords:** anomaly detection, autoencoder, variational autoencoder (VAE), long short-term memory (LSTM), attention module

## Abstract

Today, accurate and automated abnormality diagnosis and identification have become of paramount importance as they are involved in many critical and life-saving scenarios. To accomplish such frontiers, we propose three artificial intelligence models through the application of deep learning algorithms to analyze and detect anomalies in human heartbeat signals. The three proposed models include an attention autoencoder that maps input data to a lower-dimensional latent representation with maximum feature retention, and a reconstruction decoder with minimum remodeling loss. The autoencoder has an embedded attention module at the bottleneck to learn the salient activations of the encoded distribution. Additionally, a variational autoencoder (VAE) and a long short-term memory (LSTM) network is designed to learn the Gaussian distribution of the generative reconstruction and time-series sequential data analysis. The three proposed models displayed outstanding ability to detect anomalies on the evaluated five thousand electrocardiogram (ECG5000) signals with 99% accuracy and 99.3% precision score in detecting healthy heartbeats from patients with severe congestive heart failure.

## 1. Introduction

**Figure 1:**
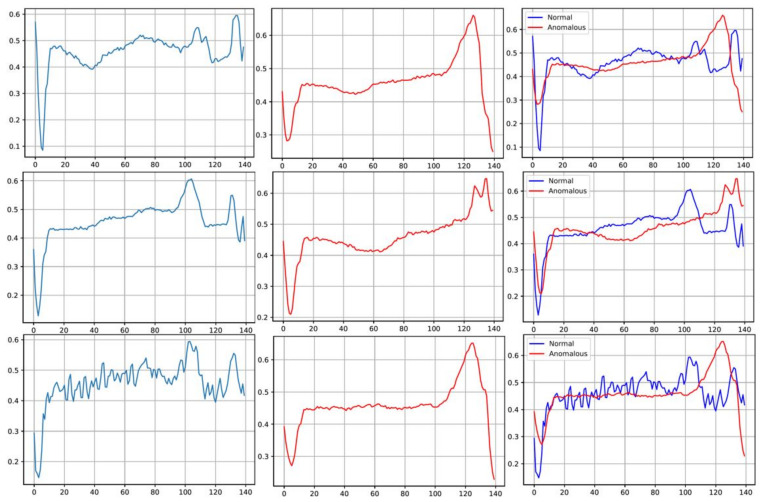
Temporal distribution of normal and abnormal heartbeat from the ECG5000 dataset. **Left column**: Normal heartbeat; **Middle column**: Anomalous heartbeat; **Right column**: Combination of normal and anomalous heartbeats.

Intelligent fault diagnosis (IFD) has presently been upheaved into an integral area of interest for most life sectors, including finance, military, healthcare, cybersecurity, and the fashion industry [1]. Primarily, fault diagnosis entails the use of complex algorithms and models to detect anomalies or abnormalities in data. For instance, in Figure 1, a typical illustration of a normal and abnormal time-series distribution sample is displayed, and it is the task of an effective anomaly detector to identify the differences [2]. In most cases, anomaly detection is framed as a data-driven approach where the fault diagnosis model applies various analytical principles to analyze and extract intricate data features for observatory purposes. As such, data points are classified with a certain degree of deviation as anomalies.

Due to the indispensability of error occurrence in most life applications, the automation of fault diagnosis mechanisms or design of intelligent anomaly detection models is beginning to observe prominent attention and play a vital role in most applications. For example, in the healthcare industry, intelligent anomaly detection systems are used for medical diagnoses, such as in moderate traumatic brain injury (mTBI) via magnetoencephalography (MEG) [3]. Furthermore, in some military applications, such as in advanced missile aircraft, abnormality detection models are implemented to diagnose and investigate fault conditions [4]. Likewise, several fraud-detection models are applied in the finance sector to instantaneously recognize deceptive transactions.

In this paper, we explore the use of machine learning and deep learning algorithms [5,6] to intelligently learn the distribution of anomalies in heartbeat signals to achieve an automated process of heart defect detection via signal sensing from the ECG. The application of machine learning algorithms, particularly the artificial neural network, is employed to detect abnormal heartbeats or rhythms in electrocardiogram (ECG) data. There are three different anomaly detectors presented in this work.

This first model which implements the autoencoder design learns the most suitable pattern to map the latent representation of input data with the maximum amount of information retained and the reconstruction of the features with the minimum reconstruction loss. The autoencoder model consists of an encoder and a decoder [7], whereby the encoder’s task is to learn the encoding of the latent-space representation of input data. Then, the decoder builds a reconstruction of the learned feature representation. Before the decoder, an attention module is designed to selectively enhance the important features of the latent vector, producing a context vector which would be fed to the decoder. This allows the decoder to concentrate on the attended vector, resulting in a comparatively small reconstruction loss compared to the original data.

The second model employs the variational autoencoder (VAE) whose decoder samples parameterize distribution from the bottleneck for complex generative modeling. The final model designs the recurrent neural network’s (RNN) long short-term memory (LSTM) architecture to process the input data as a time-series sequence. Unlike the initial two models, which serve as a reconstructive or generative model [8], the LSTM network processes the heartbeat data as a sequence, learning the saliency activations for the direct classification of the heartbeat as anomalous or healthy. Therefore, the ideas presented in this work are summarized as follows:

1. This paper proposes combining a dual network of encoder and decoder into an autoencoder to model the latent-space representation of data and its reconstruction for abnormality detection.
2. The proposed model applies Luong’s concatenation attention [9] to the autoencoder’s low-dimensional bottleneck, prompting extensive focus on specific complex data points and saliency activations into a fixed-size context vector.
3. The VAE model is applied to normalize the mean and standard deviation of the encoded Gaussian distribution for the decoder’s generative reconstruction.

**Figure 2:**
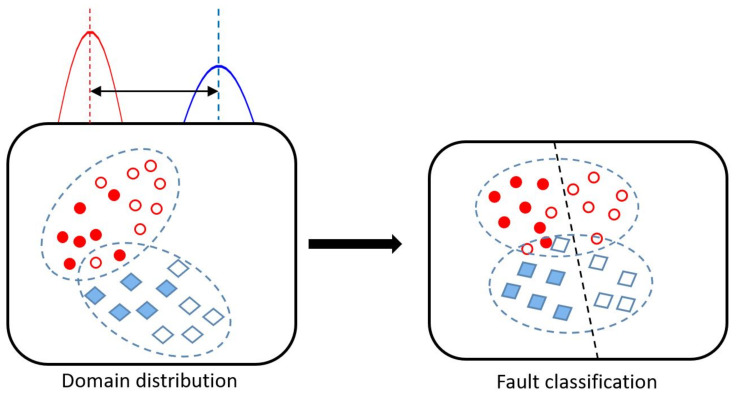
Spatial distribution of data points outlier and latent representation visualization.

Unlike other machine learning domains, such as classification or regression, where models merely use loss or cost functions to only systematically determine the direction of parametric values update [10], the error values in the autoencoder and variational autoencoder designed in this work are also utilized to interpret the representational margin where the reconstructed signals are indeed correct or anomalous. Therefore, both models should relatively result in slight losses when the input data is good, but huge losses when the data is abnormal. Such variation and representational margin in data distribution are represented in Figure 2.

Overall, the remaining components of this paper are organized as follows: the background summary is discussed in Section 2, while Section 3 presents the architectural description of the proposed models. Finally, the analyses and experimental implementation of the models are discussed in Section 4 and Section 5 concludes the work.

## 2. Related Work

### 2.1. Anomaly Detection

A simple demonstration of abnormality detection is the use of an anomaly score which measures the degree of outliers in a given dataset [11]. However, because the concept of similarity and dissimilarity varies in applications and different contexts, the notion of anomaly score is largely domain-specific.

In unsupervised scenarios, anomalies are detected using algorithmic designs which score data points based on their structural properties such as density, variance, and distance evaluation [12]. For example, the local outlier factor algorithm (LOF) applies a degree of outliers to data based on its density, therefore measuring the degree of isolation of data points in comparison to neighboring points [13]. LOF achieves this by introducing a MinDist (k) parameter representing neighboring data in a particular region of consideration. In several other clustering algorithms such as K-means and fuzzy C-means, different techniques such as Euclidean distance or squared Euclidean distances [14] are established to compute the distance between data points [15].

### 2.2. Machine Learning Models

The support vector machine (SVM) technique, which finds a hyperplane separating categories of data, was designed by Maimon et al. [16]. Their method applies the data position on the hyperplane as the class category of such data point. In addition, a kernel SVM was introduced to map data with multidimension by extending the SVM hyperplane method to fit data with a larger feature space.

Similarly, advanced artificial neural network architecture such as convolutional neural networks (CNN) and recurrent neural networks (RNN) are also employed to detect anomalies due to their vast capability to learn useful information in data. For example, with CNN, the spatial dependencies of such data are captured, such as in Rajpurkar et al. [17]. The long short-term memory (LSTM) network was also applied to analyze the normality of time-series data, taking advantage of RNN’s ability to learn temporal dependencies efficiently [18]. Additionally, the LSTM network can model a longer data sequence, applying cell states and gated outputs to retain useful information, and let go of unnecessary ones [19]. Such application is often extended to analyze intrusion detection systems or anomaly detection in network traffic [20].

Khorram et al. developed an end-to-end convolutional recurrent neural network (CRNN) to detect the fault in time-domain features collected from accelerometers [21]. This was accomplished by passing output from one architecture to another for further analysis. Furthermore, a CRNN model where the data features are first filtered out with a CNN and passed to a four-layer RNN for context understanding was proposed by [22].

### 2.3. Autoencoders

Deep learning dimensionality reduction techniques such as generative adversarial network (GAN) [23] and variational autoencoder (VAE) [24] have become very effective in modeling or mapping input data into lower dimensional distribution embedded with useful latent space representation capable of being reconstructed into quality transformations. For example, a sequence-to-sequence model named TimeNet successfully extracted sequence features automatically from time-series data via the use of a supervised autoencoder classifier for anomaly classification [25].

While a traditional autoencoder includes an encoder and a decoder, Jia et al. [26]. proposed a stacked autoencoder with several layers of encoders and decoders connected to maximize the network’s capability to extract complex patterns and features. In addition, the stacked autoencoder and stacked denoising autoencoder technique applied to the fault diagnosis of bearings presented an improved classification performance compared to the classical artificial neural network model, emphasizing the ability of the autoencoder to generalize the learned latent space representation [27].

Using a deep coupling autoencoder (DCAE) model, fault diagnosis of rotating machinery is achieved through coupling dual hidden representations from captured joint information of several multimodal sensory data and at the higher level [28]. In addition, the effectiveness of different activation functions and their impacts on diagnosis performance using an autoencoder was investigated by Shao et al. [29]. They also tested the Gaussian wavelet model as an activation function for a wavelet autoencoder with a significant improvement [30].

## 3. Attention Autoencoder Latent Representational Model

### 3.1. Concat Attention Autoencoder (CAT-AE)

**Figure 3:**
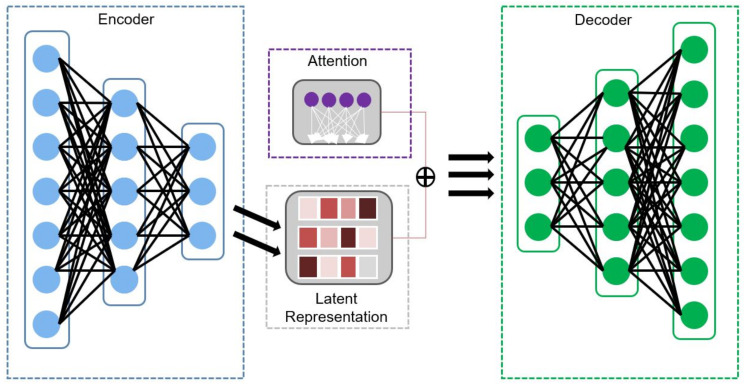
Architecture of the autoencoder model with the encoder, attention module, and the decoder.

As represented in Figure 3, the concatenation autoencoder model consists of an encoder, an attention module [9], and a decoder based on fully connected layers of feedforward artificial neural network. The encoder encodes input representation to a latent space and is then reconstructed by the decoder to the input dimension [31]. As such, the two parts of the autoencoder are expressed in Equations (1) and (2) as

(1) ϕ:χ→Z

(2) $$\psi :Z\to \ddot{\chi }$$

where ϕ is the encoder, χ is the input data, *Z* is the obtained latent representation of the encoder, ψ is the decoder, and $\ddot{\chi }$ is the reconstructed output.

#### 3.1.1. Encoder

The encoder learns the input data dimension compression to encode the features’ latent representation, whereas the decoder recreates the encoded latent representation to a reconstructed output. In the model design, the encoder is a stacked layer of three dense networks with 32, 16, and 8 neurons, respectively. Each of the layers is activated with a rectified linear unit (ReLU) [32], and the first layer is then represented in Equation (3), while the last layer is represented in Equation (4).

(3) $$Z_{1}=\sigma \left(W_{1}*x_{1}+b_{1}\right)$$

(4) $$Z_{3}=\sigma \left(W_{3}*\left[\sigma (W_{2}*\left[\sigma \left(W_{1}*x_{1}+b_{1}\right)\right]+b_{2}\right]+b_{3}\right)$$

where *x* is the input data, *W* and *b* are learnable parameters, σ represents activation function, and $Z_{3}$ is the final latent output.

#### 3.1.2. Attention Mechanism

The attention module applies a probability function to estimate scores from the bottleneck vector representation. The estimated score is multiplied by the bottleneck vector representation to obtain the context vector. Then, the context vector is fed to the decoder as the input representation, which is reconstructed to the original encoder input data dimension. Using Luong’s concatenation multiplicative technique [9], the attention score is obtained with a single neural network layer $e_{ij}$, which is calculated in Equation (5) as

(5) $$e_{ij}=W*Z+b$$

where $e_{ij}$ is the neural network output, *W* and *b* are learnable parameters, and *Z* is the latent representation. With this, the score value σ is obtained from the sigmoid function applied in Equation (6).

(6) $$\sigma_{ij}=\frac{1}{1+exp^{-x}}$$

The value scores σ are then multiplied with the encoder’s latent output, as computed in Equation (7).

(7) $$C_{t}=\sum_{j=1}^{n}\sigma_{ij}*Z_{j}$$

where $C_{t}$ is the final attention context vector and $Z_{j}$ is the encoder’s latent representation.

#### 3.1.3. Decoder

Similarly, the decoder that reconstructs the latent representation has three dense networks with 16, 32, and 140 neurons. The first two layers are activated using a rectified linear unit, while the last layer has a sigmoid activation to compute probability distribution between zero and one. The first layer is expressed in Equation (8), whereas the last layer is expressed in Equation (9).

(8) $$O_{1}=\sigma \left(W_{1}*C_{t}+b_{1}\right)$$

(9) $$O_{3}=\sigma \left(W_{3}*\left[\sigma (W_{2}*\left[\sigma \left(W_{1}*O_{1}+b_{1}\right)\right]+b_{2}\right]+b_{3}\right)$$

where $C_{t}$ is the context vector, *W* and *b* are learnable parameters, σ represents activation function, and $O_{3}$ is the final decoder reconstructed output.

#### 3.1.4. Loss Function

Having completed the model’s forward computation with the input data, the output prediction is compared with the input label, and the difference is calculated with the mean absolute error (MAE) [33] method. The loss allows the model, via backpropagation, to update the model’s parameters such that the subsequent predictions head towards the right direction and achieve improved outputs. The MAE cost function is computed as represented in Equation (10):

(10) $$MAE=\frac{1}{n}\sum_{i=1}^{n}\left|y_{i}-x_{i}\right|$$

where MAE is the summation of the absolute difference of the model prediction $x_{i}$ and the true value $y_{i}$ for all the data points *n*.

### 3.2. Variational Autoencoder (VAE)

Similar to an autoencoder, VAEs consist of an encoder and decoder but with a regularized encoding distribution at the bottleneck [34]. This ensures that the model training is confined to avoid overfitting and enable a good latent space distribution for generative reconstruction. To achieve this, the encoded latent space is normalized to represent the mean and covariance matrix of the encoded Gaussian distribution. This way, the encoded distribution is enforced to the standard normal distribution of the input data. Then, the distribution is fed to the decoder for generative reconstruction such as in the autoencoder.

#### Loss Function

Computing the loss between the input data and the reconstructed VAE output, considering the random normal distribution, is carried out using the Kullback–Leibler divergence (KL) function [35]. Unlike other loss functions, the KL loss function learns the distribution’s mean and variance measure as the model is trained.

Given input data *x*, the encoder *q* with parameters θ outputs the hidden representation *z*. Then, the encoding, which is a Gaussian probability density, is denoted as $q_{\theta }\left(z|x\right)$, resulting in the mean μ and standard deviation σ of a normal distribution.

Similarly, the decoder *p* with parameters ϕ produces the reconstructed output $p_{\phi }\left(x|z\right)$. Thus, the difference between the input and reconstructed output is measured as the reconstruction log-likelihood of $p_{\phi }\left(x|z\right)$.

As a result, the loss function of the VAE is therefore calculated as the reconstruction loss or expected negative log-likelihood of the i−th data point in the first term and the *KL* divergence in the second term, as shown in Equation (11).

(11) $$l_{i}\left(\theta ,\phi \right)=-E_{z∼q\theta }\left(z|x_{i}\right)\left[logp_{\phi }\left(x_{i}|z\right)\right]+KL(q_{\theta }\left(z|x_{i}\right)\left|\right|p_{\phi }\left(z\right))$$

### 3.3. Reconstructional Anomaly Detection

First, the anomaly detector is trained on only the normal heartbeat sequence, framed as a regression task such that the model is trained to predict continuous values rather than discrete values. Then, the loss is computed with the MAE function. Over time, the model can learn both the activation features of the normal heartbeat data and the saliency features, resulting in the reduction of the prediction error. Finally, after the model has been able to learn the right encoded latent space representation and the reconstruction for the normal heartbeat, the mean average of the error is analyzed to set an efficient threshold that distinguishes the loss distribution of normal data to anomalous heartbeat data. This threshold is set to one standard deviation above the mean loss of the trained normal data samples.

**Figure 4:**
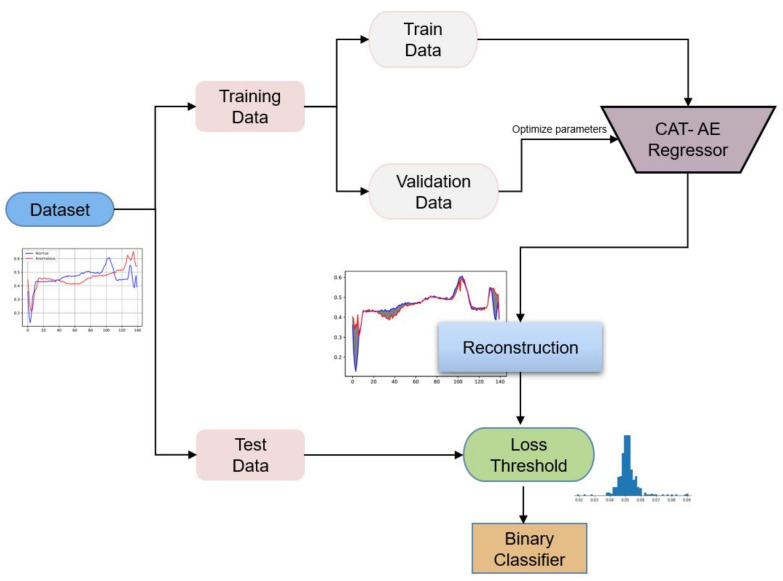
Architectural procedure for training the autoencoder model.

Secondly, the task is converted to a binary classification problem such that the obtained threshold dictates if a data is normal or abnormal by comparing its reconstruction error. Categorically, a particular input data is passed to the model, and afterwards, the reconstructed error is compared to the selected threshold. If the reconstruction error of the input data is higher than the threshold, it is classified as anomalous. For clarification, a graphical representation of the model’s algorithmic flow is presented in Figure 4.

### 3.4. Long Short-Term Memory Model

**Figure 5:**
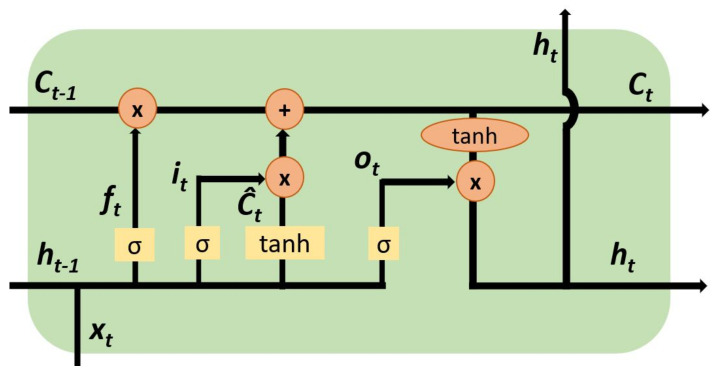
LSTM framework displaying hidden state of the time steps.

The data is framed as a time-series sequence to sequentially analyze the time steps using a gated RNN. The model is composed of two LSTM [36] layers with 100 neurons each, followed by a dropout layer neutralizing 25 percent of the neurons. Finally, there is a dense layer with one neuron which serves as the classifier with a sigmoid activator. Given the time-series data of the ECG heartbeats, the model uses the input, forget, and output gate of the LSTM to create a hidden state of the current time step, as displayed in Figure 5. This is processed with the hidden state of the previous time steps, allowing it to reserve relevant information from previous steps.

Therefore, given a previous hidden state $h_{t-1}$, and previous cell memory $c_{t-1}$ at time step *t*, the current LSTM hidden state $h_{t}$ is expressed in Equation (16) and derived from Equations (12)–(15) as

(12) $$f_{t}=\alpha \left(X_{t}*U_{f}+h_{t-1}*W_{f}\right)$$

(13) $$o_{t}=\alpha \left(X_{t}*U_{o}+h_{t-1}*W_{o}\right)$$

(14) $$I_{t}=\alpha \left(X_{t}*U_{i}+h_{t-1}*W_{i}\right)$$

(15) $$C_{t}=\alpha \left(f_{t}*c_{t-1}+I_{t}\left(tanh\left(x_{t}*U_{f}+h_{t-1}*W_{f}\right)\right)\right)$$

(16) $$h_{t}=tanh\left(C_{t}\right)*c_{t}$$

where $f_{t}$, $I_{t}$, and $o_{t}$ are forget, input, and output gates at time *t*, while *x* denotes the input data. *W* and *U* are trainable parameters, α is the nonlinear activation function, $c_{t}$ is the current cell memory, and $h_{t}$ is the current hidden state at time *t*. The model loss is computed using the binary cross-entropy function is and trained for 20 epochs.

## 4. Experimental Analysis

### 4.1. Dataset

The ECG5000 dataset is an extract from a 20 h long electrocardiogram (ECG) test [37], which records both the strength and timing of the electrical signals from the heart, mostly referred to as heartbeat. Then, the 20 h long ECG was extrapolated to an equal length of 140 points. Therefore, all of the datasets are set to a one-dimensional time-series data of 140 time steps [38]. A total of 5000 records of the extrapolated ECG were then selected to form the ECG5000 dataset, out of which 2989 are normal, and the remaining 2011 are anomalous. Therefore, the dataset has a size of 5000 × 140 dimension. The data was split into an 80:10:10 ratio for the train, validation, and test set, respectively, to train the proposed model.

### 4.2. Metrics

For the binary classifier, a true positive (*TP*) represents correctly predicted positive values, while true negative (*TN*) represents correctly predicted negative values. In addition, false positive (*FP*) denotes a positively predicted value that is negative, and false negative (*FN*) denotes a negatively predicted value that is positive. Therefore, the following metrics are implemented to evaluate the effectiveness and efficiency of all models expressed in Table 1.

1. *Accuracy*: measures how correct a model prediction is to the true label, and it is measured as the ratio of all the correctly predicted observations to the total observations, as displayed in Equation (17).
  (17) $$Accuracy=\frac{TP+TN}{TP+TN+FP+FN}$$
2. *Precision*: computes the ratio of correctly predicted positive observations to the sum of all the predicted positive observations. It measures the accuracy of the positively predicted observations shown in Equation (18).
  (18) $$Precision=\frac{TP}{TP+FP}$$
3. *Recall*: shown in Equation (19), it computes the ratio of correctly predicted positive observations to the sum of all observations classified in their true class.
  (19) $$Recall=\frac{TP}{TP+FN}$$
4. *F1 Score*: considers both the recall and precision of a model by accounting for false positives and false negatives. *F1 Score* is best suited for uneven class distribution and is shown in Equation (20).
  (20) $$F1\;Score=2\times \frac{precision\times recall}{precision+recall}$$

**Table 1:** Comparison of results performance on the ECG5000 test dataset.

Model	*Accuracy*	*Recall*	*Precision*	*F1-Score*
Hierarchical [39]	0.955	0.946	0.958	0.946
Spectral [39]	0.958	0.951	0.947	0.947
Val-thresh [40]	0.968	-	-	0.957
VRAE+Wasserstein [40]	0.951	-	-	0.946
VRAE + k-Means [40]	0.959	-	-	0.952
VAE	0.952	0.925	0.984	0.954
AE-Without-Attention	0.97	0.955	0.988	0.971
CAT-AE	0.972	0.956	0.992	0.974
LSTM	0.990	0.989	0.993	0.991

### 4.3. Training Details

First, the ECG5000 dataset is preprocessed by transforming and scaling the values which are originally between the range of −5 and 2 to 0 and 1. This normalization reduces the covariate shift and the scale of gradient parameters, resulting in accelerated training. For the CAT-AE, the normalized data is then passed to the model’s encoder. As a dimensionality reduction tool, the encoder consists of three layers that reduce the 140 input points to 8, each activated with a rectified linear unit.

The encoder generates a latent representation of the input data with a one-dimensional vector of length eight, representing the activation features of each input data. To further enrich the decoder with the most crucial input data information required to accomplish a perfect reconstruction, an attention module consisting of a single feedforward layer network with eight neurons is designed to adaptively focus on the most important cues in the latent representation. The attention layer finally generates a context vector of the data’s latent space, emphasizing the encoded saliency features of each ECG time-step record. This is achieved by implementing the concatenation technique of Luong’s attention mechanism, obtaining value scores from the latent representation via the one-layer neural network. Next, the score is converted to a normalized probability distribution, applying a weighted measure to the decoder’s reconstruction. Subsequently, the weighted probability distribution is multiplied by the original encoder output to form the attention context vector.

Finally, the attention context vector is fed to the decoder, reconstructing the ECG 140 time-step sequence. Overall, the final model prediction is compared to the real label values, and the cost is computed by employing the mean average error. Then, the loss derivative is computed using backpropagation techniques, and the gradients are optimized with the Adam optimizer. The model was trained for 15 epochs, resulting in the optimal result with a batch size of 512 on each iteration. After the 15th epoch, the model loss was drastically reduced to around 0.0241.

To achieve the classification of the normal and anomalous heartbeats, the CAT-AE reconstruction loss is compared to the original data distribution to determine the distinguishing threshold. The threshold implemented in this study is set to the mean of the reconstructed loss plus one standard deviation of the mean.

As such, the training process updates the encoder’s weights to generate the optimal latent representation of the input data while the attention weights are updated to detect the salient activations of the latent features. Finally, the decoder weights are updated to reconstruct the context vector generated by the attention module.

For the VAE model, aside from the input and output layers with 140 units, both the encoder and decoder have one hidden layer with 32 neurons, while the bottleneck, which computes the mean and variance, has two neurons each. The model loss is computed with the KL function and is trained for 100 epochs. All of the other parameters are the same as the CAT-AE model, whereas for the LSTM classifier network, the binary cross-entropy function computed the loss and trained for 20 epochs with the Adam optimizer.

### 4.4. Experimental Results

#### 4.4.1. CAT-AE

As shown in Table 1, the proposed CAT-AE model outperformed several of the state-of-the-art models in all evaluated metrics. This displays the model’s ability to efficiently learn the latent representation of the ECG data, the saliency features, and the proper reconstruction to generate the right time-series distribution. In addition, the model can establish an excellent feature learning with the attention module focusing on the most significant points of the time steps.

For example, compared to the hierarchical model of [39], the CAT-AE model recorded an increase of 1.75% and 1.05% accuracy and recall scores. The increased accuracy score indicates that the generally correctly predicted values are more in the CAT-AE model, and also, the recall score depicts that the correctly predicted positive observations are also improved in the CAT-AE model. Similarly, a 3.43% and 2.88% increment were measured in the precision and F1 score of the CAT-AE model in comparison to the hierarchical model of [39].

Compared to VRAE with Wasserstein distance (VRAE+ Wasserstein) [40], the CAT-AE achieved better results with 2.16% and 2.87% increment in the accuracy and F1 score, respectively. In addition, a 1.34% and 2.26% increase were recorded in the accuracy and F1 score compared to the VRAE with means clustering (VRAE+ K-means) [40]. This shows that the positive and negative observation predictions were correctly identified with the CAT-AE model, emphasizing the model’s ability to model the right reconstruction parameters for the ECG5000 dataset. Furthermore, the CAT-AE also shows refined performance against the spectral model, with a 4.54% and 2.77% upturn for the precision and F1 score values. This is also accompanied by a difference of 1.44% and 0.52% in accuracy and recall score, with CAT-AE showing superiority.

#### 4.4.2. AE without Attention

Though the autoencoder designed in this study is suitable for learning the latent representation distribution of the ECG5000 dataset, we display the effectiveness of the concatenation attention by comparing the CAT-AE model to the model without the attention module (AE-No-Attention). Comparing both models with the evaluated metrics indicates that the attention module significantly boosts the model’s ability to learn and focus on the saliency representation. The CAT-AE model surpasses the AE-No-Attention with 0.21% accuracy and 0.12% recall. This indicates that the attention mechanism plays a role in identifying more correctly predicted observations. In addition, the CAT-AE model surpasses the AE-No-Attention with a 0.40% precision score and 0.31% F1 score, respectively.

#### 4.4.3. LSTM

**Figure 6:**
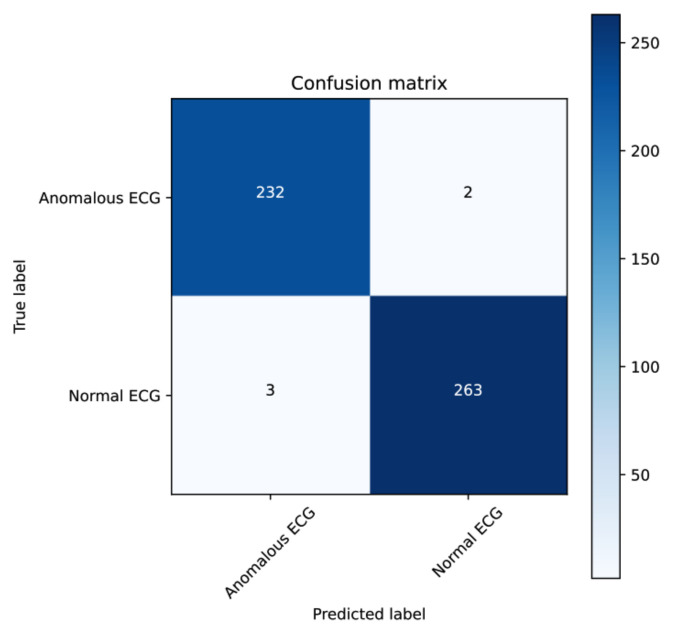
Confusion matrix of the LSTM-predicted result on the test set.

The LSTM model records the best results compared to the other models. For the precision metrics, the CAT-AE model recorded 99.2% compared to the LSTM’s 99.3%. Similarly, LSTM has a higher score than the others in all the remaining metrics. In contrast to the VAE model, LSTM has an improved accuracy score of 3.84% and recall improvement of 6.47%. In addition, it proves to be superior with an F1 score of 3.74%. Compared to the CAT-AE, LSTM also has a greater accuracy of 99%, while the CAT-AE model has 97.2%. The LSTM model proves to be more suitable for time-series sequential data as it processes the heartbeat signals at different time steps, a robust advantage of the recurrent network. This is emphasized in the confusion matrix per class representation in Figure 6, which summarizes the predicted count values for the classes. Notwithstanding, the CAT-AE proved to be more constructive in developing a reconstruction of the input data from learned latent space, and the VAE architecture has proved excellent in the literature at extending a reconstruction to new samples of data. The valuation of the models on the validation set are also presented in Table 2.

## 5. Conclusions

In this article, a concatenation attention autoencoder (CAT-AE), variational autoencoder, and LSTM time-series models are proposed to detect anomalies in heartbeat sequences from ECG data. The ECG data contains heartbeat sequences of healthy individuals and patients with severe congestive heart failure, analyzed using machine learning algorithms. The first model, CAT-AE, follows the autoencoder design for learning the latent representation of the input heartbeat sequence with an attention mechanism and then reconstructs the learned features with the slightest information lost and error. In addition, the VAE model maps the generative reconstruction of the data via a Gaussian distribution of the latent space’s mean and standard deviation. Lastly, the LSTM network models the data sequence using a gated hidden state to learn the essential time-step features for detecting anomalies in the ECG heartbeats.

Evaluated on the ECG5000 heartbeat dataset, the presented models achieved outstanding anomaly detection capability with state-of-the-art outcomes. Similarly, the CAT-AE’s encoder reveals exceptional ability to learn the reduced dimensional salient features of the ECG data, crucial for detecting the reconstruction loss threshold and accurate classification of the input data. Furthermore, the LSTM network’s supremacy, with 98.9% recall and 99% accuracy, shows the excellent ability of the model to determine abnormality in the ECG heartbeat signals.

## Figures and Tables

**Table 2:** Comparison of results performance on the ECG5000 validation set.

Model	*Accuracy*	*Recall*	*Precision*	*F1-Score*
VAE	0.948	0.932	0.978	0.954
AE-Without-Attention	0.956	0.950	0.970	0.960
CAT-AE	0.958	0.946	0.977	0.946
LSTM	0.984	0.998	0.973	0.986

## Data Availability

The ECG5000 Data is available at http://www.timeseriesclassification.com/description.php?Dataset=ECG5000 (accessed on 10 December 2021).
